# Eating behaviors in obese children with pseudohypoparathyroidism type 1a: a cross-sectional study

**DOI:** 10.1186/1687-9856-2014-21

**Published:** 2014-10-15

**Authors:** Lulu Wang, Ashley H Shoemaker

**Affiliations:** 1 School of Medicine, Vanderbilt University, Nashville, TN, USA; 2 Monroe Carell Jr. Children’s Hospital at Vanderbilt University, Nashville, TN 37232-9170, USA

**Keywords:** Pseudohypoparathyroidism, Obesity, Eating behaviors

## Abstract

**Background:**

Children with pseudohypoparathyroidism type 1a (PHP-1a) develop early-onset obesity. These children have decreased resting energy expenditure but it is unknown if hyperphagia contributes to their obesity.

**Methods:**

We conducted a survey assessment of patients 2 to 12 years old with PHP-1a and matched controls using the Hyperphagia Questionnaire (HQ) and Children’s Eating Behavior Questionnaire (CEBQ). Results of the PHP-1a group were also compared with an obese control group and normal weight sibling group.

**Results:**

We enrolled 10 patients with PHP-1a and 9 matched controls. There was not a significant difference between the PHP-1a group and matched controls for total HQ score (p = 0.72), Behavior (p = 0.91), Drive (p = 0.48) or Severity (p = 0.73) subset scores. There was also no difference between the PHP-1a group and matched controls on the CEBQ. In a secondary analysis, the PHP-1a group was compared with obese controls (n = 30) and normal weight siblings (n = 6). Caregivers reported an increased interest in food before age 2 years in 6 of 10 PHP-1a patients (60%), 9 of 30 obese controls (30%) and none of the siblings (p = 0.04). The sibling group had a significantly lower Positive Eating Behavior score than the PHP-1a group (2.6 [2.4, 2.9] vs. 3.5 [3.1, 4.0], p < 0.01) and obese controls (2.6 [2.4, 2.9] vs. 3.4 [2.6, 3.8], p = 0.04), but there was not a significant difference between the PHP-1a and obese controls (p = 0.35). The sibling group had a lower Desire to Drink score than both the PHP-1a group (1.8 [1.6, 2.7] vs. 4.3 [3.3, 5.0], p < 0.01) and obese controls (1.8 [1.6, 2.7] vs. 3.3 [3.0, 4.0], p < 0.01) but there was not a significant difference between the PHP-1a and obese control Desire to Drink scores (p = 0.11).

**Conclusions:**

Patients with PHP-1a demonstrate hyperphagic symptoms similar to matched obese controls.

## Introduction

Pseudohypoparathyroidism type 1a (PHP-1a) is a rare disorder caused by a maternally inherited mutation in the gene *GNAS. GNAS* encodes the alpha subunit of the stimulatory G-protein (G_s_α). In some tissues, the paternal allele is imprinted and only the maternal allele is expressed. Therefore, in imprinted tissues, patients with PHP-1a lack functional G_s_α and have abnormal G-protein coupled receptor signaling. Examples of known imprinted tissues include kidney, thyroid, hypothalamus and pituitary [1-3]. PHP-1a is usually diagnosed in childhood due to a distinctive phenotype that includes short stature, brachydactyly, cognitive impairment, ectopic ossifications and multi-hormone resistance.

A more recently described feature of PHP-1a is early-onset obesity [4]. This obesity occurs despite adequate treatment of hormone deficiencies, including thyroid hormone and growth hormone replacement. A current hypothesis is that the obesity phenotype is due to abnormal function of the melanocortin-4 receptor (MC4R) in the hypothalamus. The MC4R is a G-protein coupled receptor that plays a critical role in energy homeostasis. Mutations in *MC4R* are the most common cause of monogenic obesity in humans [5].

In a previous study, we showed that children with PHP-1a have decreased resting energy expenditure which may contribute to the obesity phenotype [6]. Hyperphagia is seen in humans and mice with abnormal MC4R signaling [5,7] but has not been demonstrated in the PHP-1a mouse model [1]. It is not known if hyperphagia or abnormal eating behaviors are seen in children with PHP-1a. We hypothesized that children with PHP-1a have mild hyperphagia leading to excess caloric intake and abnormal weight gain. To test this hypothesis, we measured eating behaviors and hyperphagia in children with PHP-1a and matched controls.

## Methods

### Participants

Study participants were recruited from the Vanderbilt adult and pediatric endocrinology clinics as well as online advertisements from August, 2012 through January, 2014. Inclusion criteria were age 2–12 years old, English proficiency and one of the following: clinical diagnosis of PHP-1a or status as a sibling of a PHP-1a patient. Exclusion criteria were: obesity due to another known genetic syndrome (e.g., Prader-Willi syndrome) or current use of appetite-suppressing medications.

Healthy, matched controls were recruited from the Vanderbilt pediatric clinics and the Vanderbilt Childhood Obesity Registry (VCOR). VCOR is a registry for patients with a history of BMI >97^th^ percentile before 6 years old. Controls were matched based on gender, age (±2 years) and BMI Z-score (±0.25). All patients in VCOR age 2–12 years old were included in the general obese control group.

Consent and age appropriate assent were obtained. The study was approved by the Institutional Review Board of Vanderbilt University.

### Experimental procedure

All participants completed a medical history form. Children’s height and weight were obtained from the most recent endocrinology or primary care clinic visit. BMI height and weight z-scores were calculated as standard deviations from the mean using gender and age specific Centers for Disease Control growth charts. All children had a Hyperphagia Questionnaire (HQ) [8] and Children’s Eating Behavior Questionnaire (CEBQ) [9] completed by the primary caregiver.

The HQ was originally developed to assess hyperphagia in Prader-Willi syndrome and contains 11-questions that assess symptoms of hyperphagia in one of three categories (Behavior, Drive and Severity), as rated on a five-point scale (1 = not a problem to 5 = severe and/or frequent problem). The total HQ score has a minimum of 11 points and a maximum of 55. The 35-item CEBQ assesses Positive and Negative Eating Behaviors. The Positive Eating Behavior score is comprised of four sub-scales: Food Responsiveness, Enjoyment of Food, Emotional Overeating and Desire to Drink.

### Data collection

Study data were collected and managed using REDCap electronic data capture tools hosted at Vanderbilt University. REDCap (Research Electronic Data Capture) [10] is a secure, web-based application designed to support data capture for research studies. Questionnaires were completed either online (REDCap survey) or via mail.

### Statistical analysis

The primary outcome was the subset scores of the HQ in PHP-1a patients compared with matched controls. Secondary analyses include comparison of CEBQ subset scores and comparison of PHP-1a, sibling and obese control groups. Results are presented as median (interquartile range) unless otherwise specified. Paired analysis was done using Wilcoxon signed rank test. For the secondary analysis of the three groups (PHP-1a, obese controls and sibling controls), continuous variables were compared using the Kruskal-Wallis test. If the p value was <0.05, each group was compared using the Mann–Whitney U test. Categorical variables were compared using Chi-square test. Based on our sample size, we did not use regression or analyses of covariance due to concerns for over-fitting. Internal consistency of the HQ was assessed using Cronbach’s alpha. Statistics were performed using SPSS version 22.

## Results

### Study population

Ten patients with PHP-1a enrolled in the study, including 2 sibling pairs. We identified a matched control for 9 of 10 PHP-1a patients. We enrolled all unaffected siblings (n = 6) to serve as a normal weight control group. The two PHP-1a sibling pairs and one additional PHP-1a subject did not have an unaffected sibling. An additional 21 obese children between 2 and 12 years old had questionnaire data available in VCOR. These children were combined with the matched controls to form an obese control group (n = 30) for secondary analysis.

### Baseline characteristics

Patients were matched on age, gender and BMI z-score (Table 1). All PHP-1a patients were white and 6 of 9 matched controls where white, the remaining patients were African American. Table 2 summarizes the baseline characteristics of the subjects. The PHP-1a group age range was 2 to 10 years old, the obese control group was 3 to 12 years old and the sibling control group was 3 to 12 years old. As expected, the PHP-1a and obese control groups were significantly more obese than the sibling controls as measured by BMI z-score (p <0.01) but there was no difference between the PHP-1a and obese control groups (p = 0.86). The obese control group was 57% white and 43% African American versus the PHP-1a group that was 100% white (p = 0.02).

**Table 1:** Baseline characteristic of the matched pairs

**Patient**	**PHP-1a group**		**Matched control**	
	**Age**	**BMI z-score**	**Age**	**BMI z-score**
1	2	3.33	---	---
2	3	1.71	3	1.54
3	4	1.16	4	1.08
4	5	2.78	7	2.72
5	5	1.44	5	1.39
6	6	2.98	5	3.01
7	9	3.01	9	2.95
8	9	2.41	10	2.44
9	10	1.85	11	1.74
10	10	2.70	10	2.63

PHP-1a, pseudohypoparathyroidism type 1a; BMI, body mass index. BMI z-scores were calculated as standard deviations from the mean using gender and age specific Centers for Disease Control growth charts.

**Table 2:** Baseline characteristics

	**PHP-1a (n = 10)**	**Obese controls (n = 30)**	**p value**	**Siblings (n = 6)**	**p value**
Age (years)	5.5 (3.8, 9.3)	7.0 (5.0-10.0)	0.22	7.5 (4.5, 11.3)	0.43
Gender (% female)	60	70	0.70	100	0.23
Ethnicity (% white)	100	57	0.02	100	1.0
BMI	24.5 (18.2, 30.5)	29.5 (21.6, 35.1)	0.43	*18.4 (14.8, 22.8)	0.05
BMI Z-score	2.55 (1.64, 2.99)	2.50 (1.74, 2.84)	0.86	*0.48 (−0.72, 1.16)	<0.01

PHP-1a, pseudohypoparathyroidism type 1a; BMI, body mass index. Data expressed as median (lower quartile, upper quartile). P-values were determined using Mann-Whitney U test and Fisher’s Exact test, comparing the PHP-1a group with each control group. BMI z-scores were calculated as standard deviations from the mean using gender and age specific Centers for Disease Control growth charts. *BMI data was not available for 2 siblings.

### Hyperphagia questionnaire

**Figure 1:**
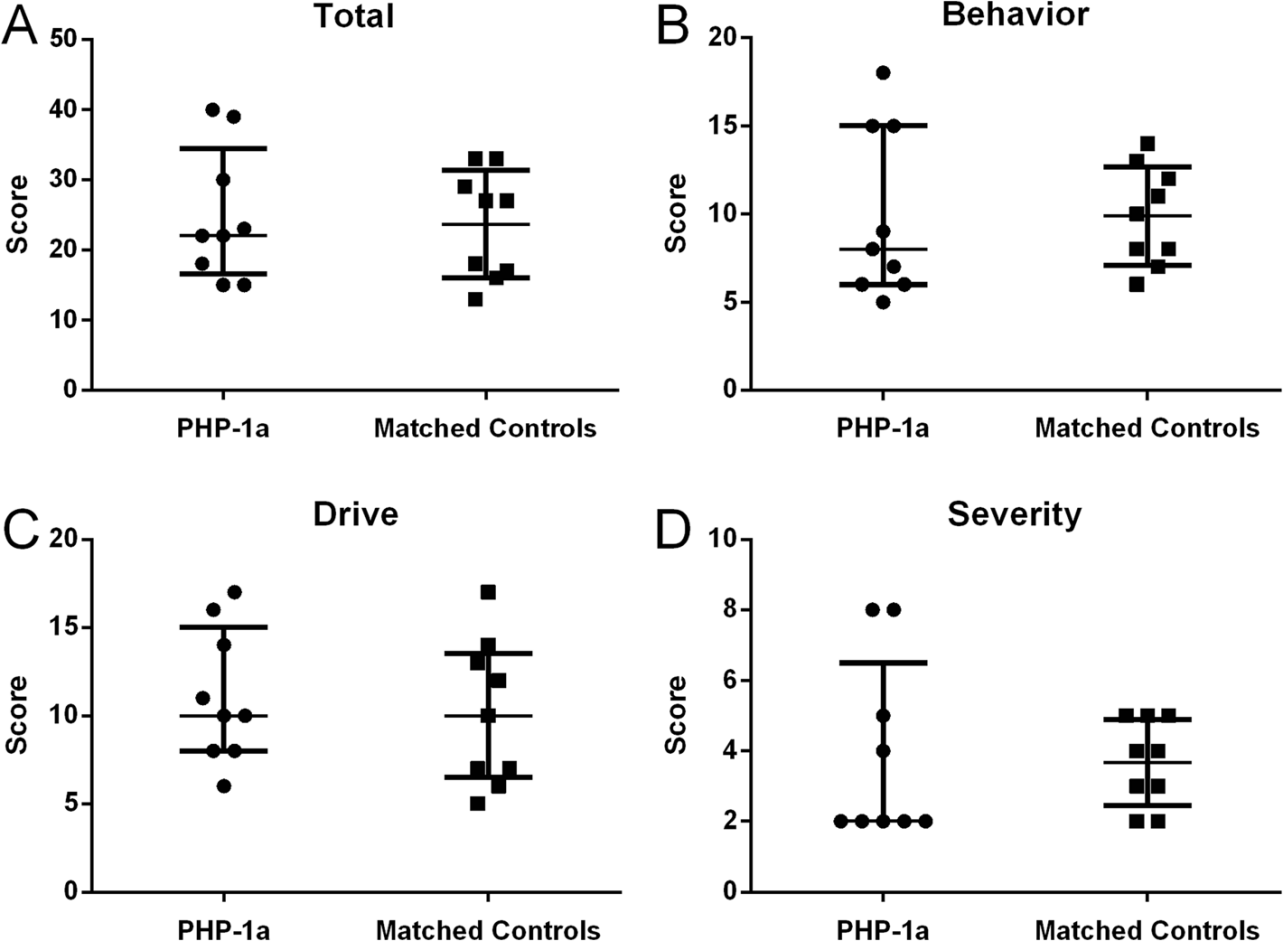
**Hyperphagia questionnaire scores, total and subset, in children with pseudohypoparathyroidism type 1a (PHP-1a, circles) and matched controls (squares).** There was no significant difference between groups by Mann–Whitney U test (Total HQ score p = 0.72, Behavior p = 0.91, Drive p = 0.48 or Severity p = 0.73). Values shown are median ± interquartile range.

**Figure 2:**
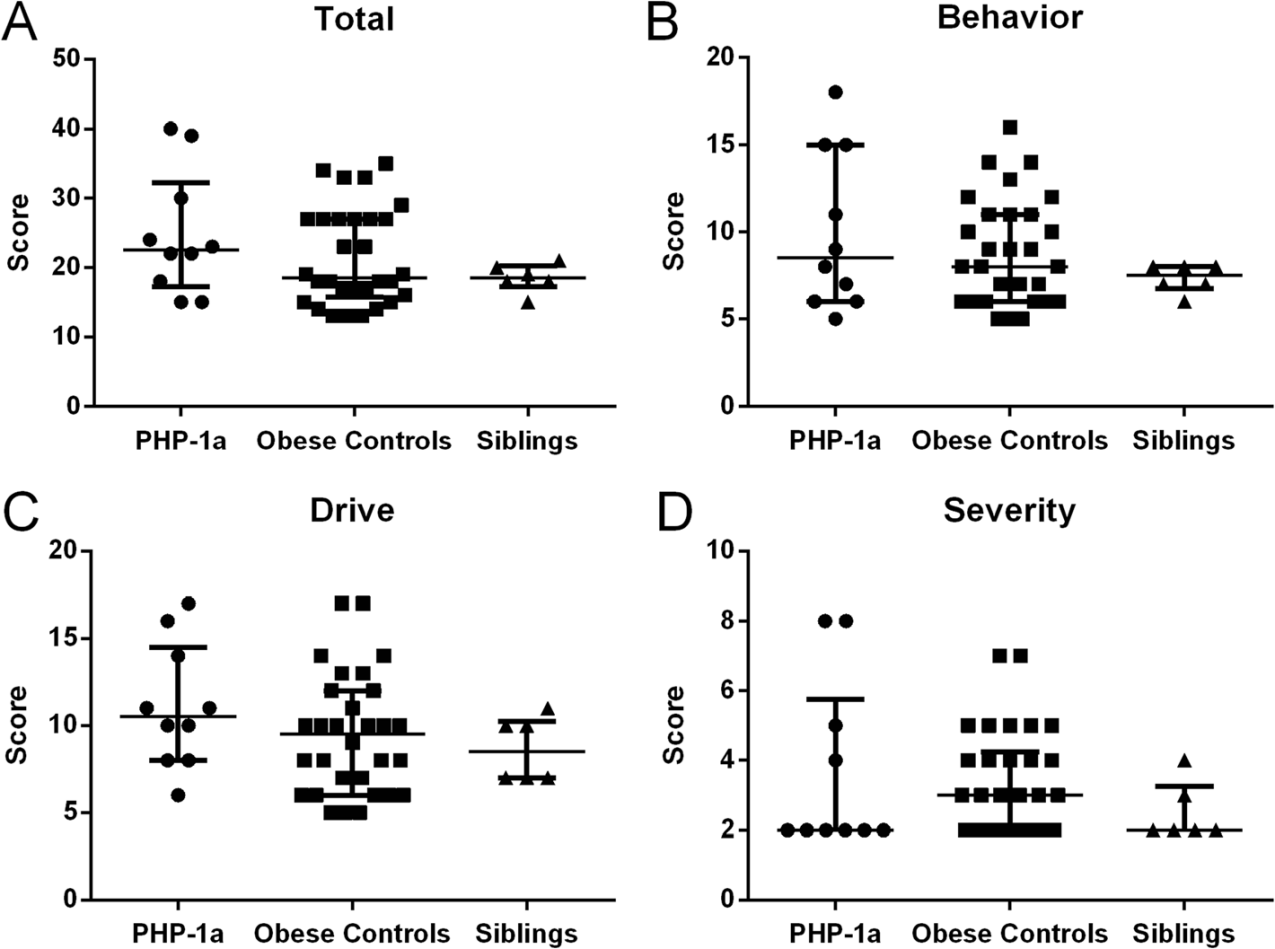
**Hyperphagia questionnaire scores, total and subset, in children with pseudohypoparathyroidism type 1a (PHP-1a, circles), obese controls (squares) and siblings (triangles).** There was no significant difference between groups by Kruskal Wallis test (Total HQ score p = 0.40, Behavior p = 0.60, Drive p = 0.31 or Severity p = 0.43). Values shown are median ± interquartile range.

There was not a significant difference between the PHP-1a group and matched controls for total HQ score (p = 0.72), Behavior (p = 0.91), Drive (p = 0.48) or Severity (p = 0.73) (Figure 1). Two PHP-1a patients were outliers with total HQ scores of 39 and 40 (group median 22.5 [17.3, 32.3]) though they were not more obese than the overall group (BMI z-score 2.78 and 2.41, group median 2.55 [1.64, 2.99]). There was also not a significant difference between the PHP-1a, obese control and sibling groups for total HQ score (22.5 [17.3, 32.3] vs. 18.5 [15.8, 27.0] vs. 18.5 [17.3, 20.3], p = 0.40) or subset scores (Figure 2). The HQ includes a question “how old was your child when they first showed an increased interest in food” that is not included in the HQ score. Caregivers reported an increased interest in food before age 2 years in 6 of 10 PHP-1a patients (60%), 9 of 30 obese controls (30%) and none of the siblings (p = 0.04). Internal reliability coefficients (Cronbach’s alpha) were 0.86 for the total HQ score, 0.64 for Behavior, 0.84 for Drive and 0.72 for Severity.

### Childhood eating behavior questionnaire

**Figure 3:**
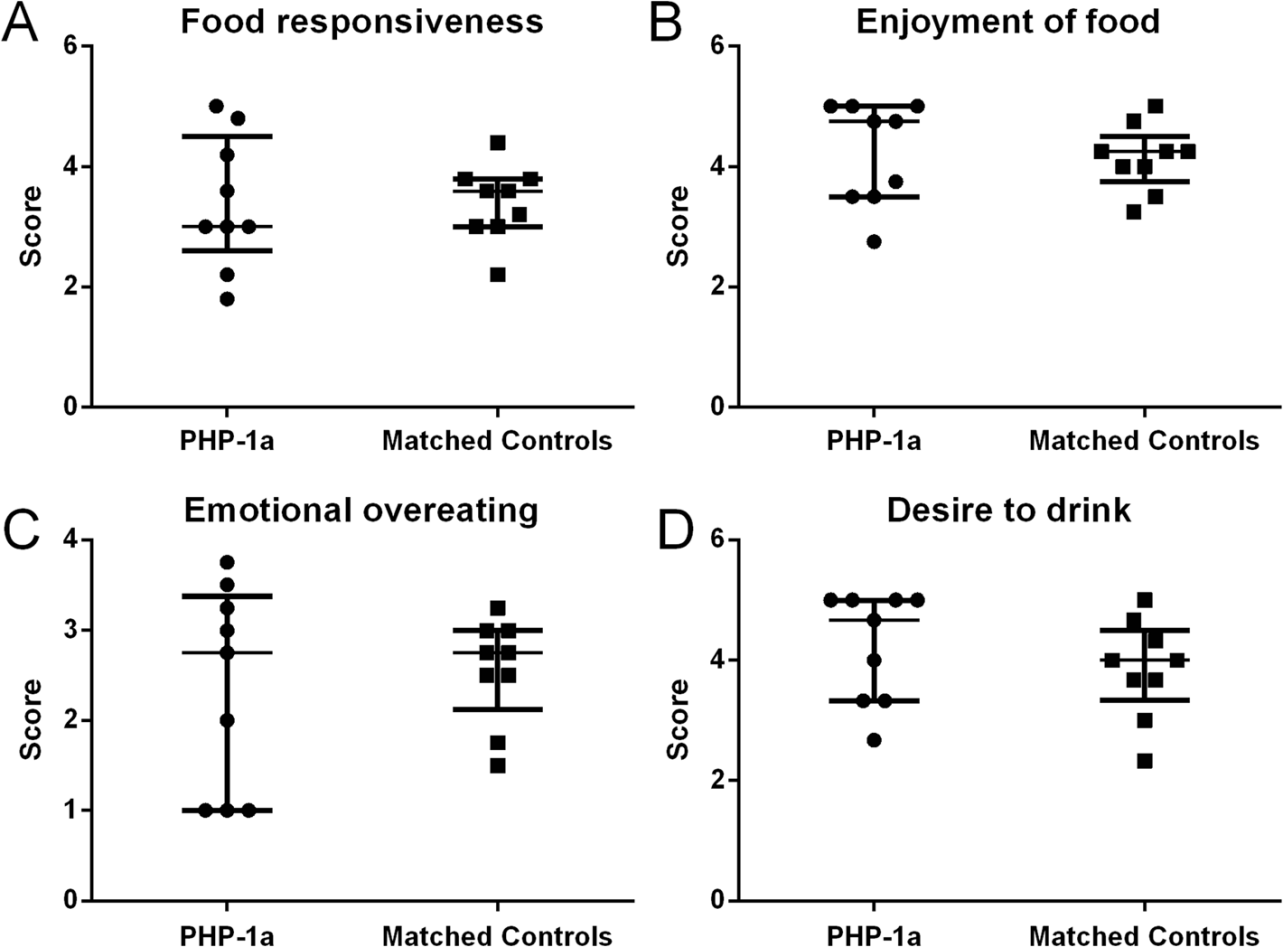
**Childhood eating behavior questionnaire positive eating behavior subset scores in children with pseudohypoparathyroidism type 1a (PHP-1a, circles) and matched controls (squares).** There was no significant difference between groups by Mann–Whitney U test (Food Responsiveness p = 1.0, Enjoyment of Food p = 0.81, Emotional Overeating p = 0.73 and Desire to Drink p = 0.31). Values shown are median ± interquartile range.

**Figure 4:**
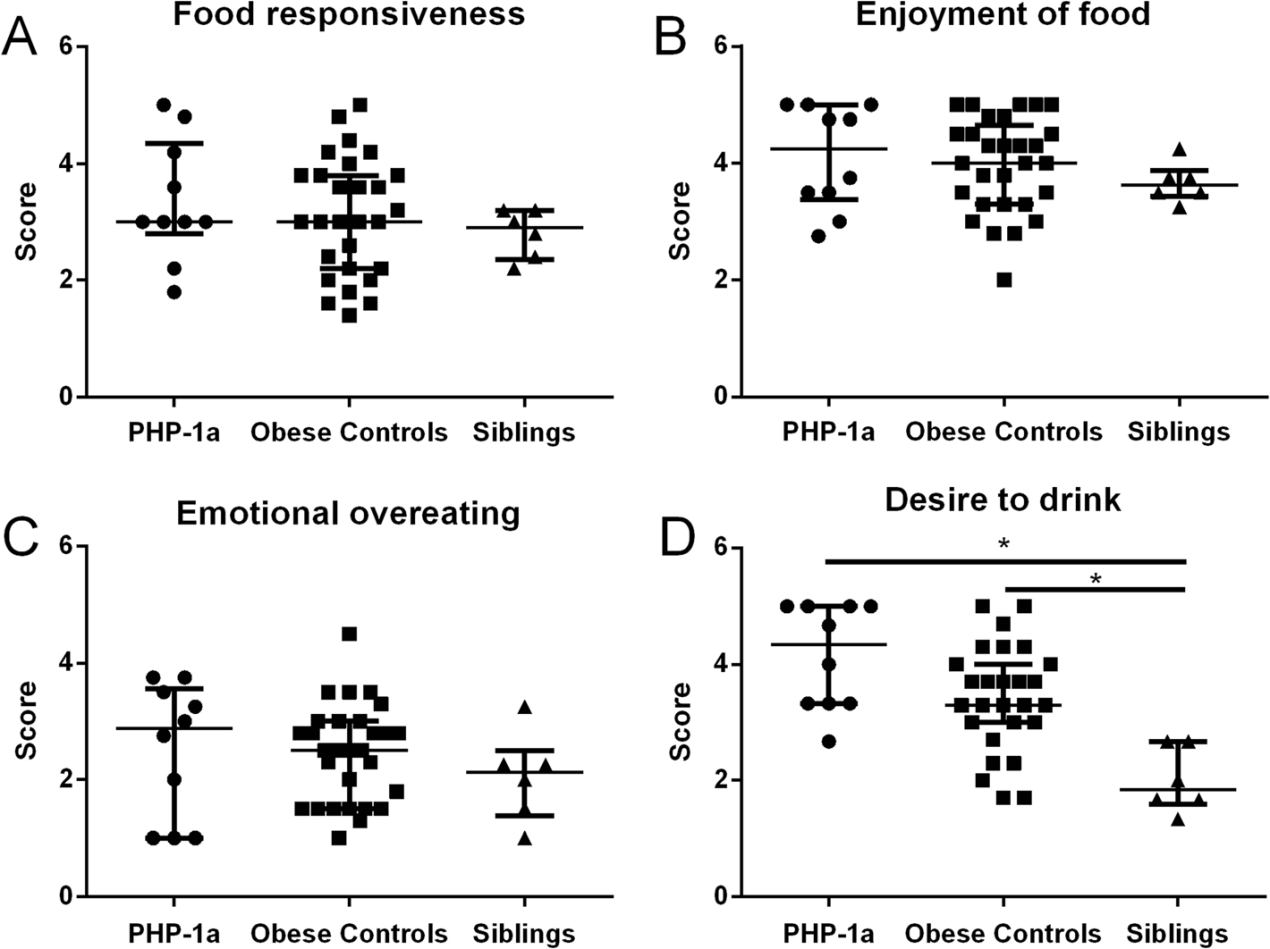
**Childhood eating behavior questionnaire positive eating behavior subset scores in children with pseudohypoparathyroidism type 1a (PHP-1a, circles), obese controls (squares) and siblings (triangles).** Values shown are median ± interquartile range. *Indicates p value <0.05 by Mann–Whitney U test following a significant Kruskal-Wallis test.

There was no significant difference between the PHP-1a group and matched controls for Negative Eating Behaviors (2.9 [2.8, 3.2] vs. 2.9 [2.6, 3.2], p = 0.77) or Positive Eating Behaviors (3.5 [2.9, 4.2] vs. 3.4 [3.1, 3.7], p = 0.86). The subscale scores for Food Responsiveness (p = 1.0), Enjoyment of Food (p = 0.81), Emotional Overeating (p = 0.73) and Desire to Drink (p = 0.31) were not different between matched pairs (Figure 3). One obese control patient did not have CEBQ data available; therefore 29 obese control subjects were included for this sub-analysis (Figure 4). The sibling group had a significantly lower Positive Eating Behavior score than the PHP-1a group (2.6 [2.4, 2.9] vs. 3.5 [3.1, 4.0], p < 0.01) and obese controls (2.6 [2.4, 2.9] vs. 3.4 [2.6, 3.8], p = 0.04), but there was not a significant difference between the PHP-1a and obese controls (p = 0.35). There was a difference between the PHP-1a, obese control and sibling groups for the Desire to Drink subscale (p < 0.01, Figure 4). The sibling group had a lower Desire to Drink score than both the PHP-1a group (1.8 [1.6, 2.7] vs. 4.3 [3.3, 5.0], p < 0.01) and obese controls (1.8 [1.6, 2.7] vs. 3.3 [3.0, 4.0], p < 0.01). There was not a significant difference between the PHP-1a and obese control Desire to Drink scores (p = 0.11).

## Discussion

To our knowledge, this is the first study to systematically evaluate eating behaviors in children with PHP-1a. While *MC4R* haploinsufficiency is associated with marked hyperphagia [5,11], our data suggest that children with PHP-1a may not be hyperphagic when compared with other obese children. Similarly, the PHP-1a mouse model shows increased feed efficiency, not increased food intake [1,2]. A recent study in adults found no difference in energy intake between PHP-1a and matched controls using 7-day food records [12]. There is one published case series of parent reported hyperphagia in two young children with PHP-1a [13]. The variability seen in PHP-1a could be due to variable imprinting of *MC4R* in the hypothalamus, or hyperphagia in *MC4R* haploinsufficiency may be mediated through non-G_s_α signaling pathways. Previously, we have demonstrated that these children have decreased resting energy expenditure [6]. This energy expenditure imbalance may be the main driver of early-onset obesity in PHP-1a.

The HQ has previously been used to evaluate eating behaviors in other forms of syndromic obesity. The HQ scores for PHP-1a (mean ± SD, total: 24.8 ± 8.9, behavior: 10.0 ± 4.5, drive: 11.1 ± 3.6, severity: 3.7 ± 2.5) were significantly lower than Prader-Willi syndrome (total: 30.5 ± 5.8, behavior: 13.6 ± 4.5, drive: 12.3 ± 3.3, severity: 4.6 ± 1.7) [8], but only slightly lower than brain-derived neurotrophic factor (*BDNF*) haploinsufficiency (total: 26.3 ± 7.3, behavior: 10.6 ± 3.2, drive: 11.5 ± 3.4, severity: 4.2 ± 1.7) [14] and Bardet-Biedl syndrome (total: 27.3 ± 8.2, behavior: 12.3 ± 3.8, drive: 11.1 ± 3.7, severity 3.9 ± 1.6) [8,15]. In contrast with PHP-1a [1,2], mouse models of Bardet-Biedl syndrome [16,17] and *Bdnf* inactivation [18] demonstrate obesity from increased food intake. It is possible that PHP-1a patients are only hyperphagic during specific developmental periods. Rapid weight gain in infancy and parent reported “increased interest in food” at a young age may indicate early, non-progressive hyperphagia in PHP-1a.

PHP-1a and obese controls had increased Positive Eating Behavior and Desire to Drink scores compared with lean siblings, but not compared with obese controls. Heightened preference for and consumption of sugar-sweetened beverages has been linked to increased Desire to Drink scores in children [19] and the motivation for drinking may be desire for the pleasant sensation of sweet taste. Alternatively, these children may demonstrate a drive for high caloric foods, aided by consumption of sugar-sweetened beverages. A reduction in the intake of sugar-sweetened beverages may be an effective strategy to reduce the rate of weight gain in obese children [20,21].

The main limitation of our study was the small sample size. PHP-1a is a rare disorder and it is challenging to identify eligible subjects. We used a matched control group in order to account for possible confounders without over-fitting our statistical model, but a matched control was not available for one child with PHP-1a. In addition, controls were not matched based on race and this may have masked differences between the groups. The HQ was originally designed for children with Prader-Willi syndrome but we believe that it is an appropriate tool for PHP-1a as both disorders cause intellectual disability, multiple medical problems and childhood obesity. An advantage of this questionnaire is that it is completed by the parent which allows the investigation of eating behaviors in young children and those with intellectual disability. The Cronbach’s alpha results for our study population (range 0.64-0.86) were all acceptable and similar to those in the original study of Prader-Willi syndrome (range 0.60-0.80) [8].

## Conclusion

In summary, our data suggest that children with PHP-1a demonstrate hyperphagic symptoms similar to other obese children. The mild hyperphagia seen in PHP-1a may be common in obese children and not attributable to PHP-1a. Further studies, such as standardized test meals and longitudinal investigations, are needed to confirm our findings. Understanding the pathophysiology of abnormal weight gain in children with PHP-1a may inform our understanding of energy homeostasis in humans and is critical in order to design targeted therapeutic interventions.

## Competing interests

The authors have no conflict of interest to disclose.

## Authors’ contributions

LW generated figures and wrote the manuscript. AHS designed the study, collected and analyzed data and assisted in writing the manuscript. Both authors read and approved the final manuscript.
